# The Ætiology of Hydrophobia

**Published:** 1910-12-17

**Authors:** 


### Medicine.

### THE ÆTIOLOGY OF HYDROPHOBIA.

In this country, owing to our insular position and a scheme of effective quarantine, rabies is now practically unknown, and research into its cause is therefore at a standstill. Abroad, however, this is not the case, and the latest investigations of Negri, published in 1909 in the *Zeitschrift für Hygiene und Infectionskrankheiten*, are being accepted as an advance of knowledge which is of permanent value. Negri has demonstrated certain bodies in the protoplasm of the nerve cells from cases of hydrophobia, which undergo a cycle of development and finally sporulate. He regards these forms as protozoal, and as the parasitic cause of rabies; and other workers seem to be coming to the conclusion that he is right (Reichel, Whitington, Frothingham). These bodies were first described by Negri in 1903; they are round, oval, or elliptical in shape, and are never found except in rabies. The name neurocytes has been given to them, since they are confined to the cells of the central nervous system. In size they vary from $2\mu$ to $8\mu$ in man; in some other animals they may be $20\mu$ or more in length; there may be more than one in a cell. Their seats of predilection are the pyramidal cells of the horn of Ammon, and the Purkinje cells of the cerebellum.

The neurocytes stain well with eosin, and Negri especially recommends Mann's and Romanowsky's stains. Many other stains will give good results, and osmic acid is said to show them up well. The structure of the Negri body is somewhat complex. Numbers of very small, round, highly refractile particles are to be seen; and there are also larger forms, round or oval in shape, less refractile, and granular in appearance, of which but one or two are contained in each neurocyte. Transition forms between these two exist. These masses their discoverer regards as chromatin. There is yet another kind of content in the neurocytes which is supposed to be connected with spore formation. The spores themselves are believed to be ultramicroscopic.

Certain criticisms on these observations were offered three years ago.* It is objected that the Negri bodies are never found in material that is surely virulent, such as the saliva, the salivary glands, and the nerves. The answer is that the virus is conveyed from patient to patient in the spore form, too minute to be detected microscopically. To this Frosch says that the virus of rabies passes through filters the pores of which are so fine as to be impervious to the smallest particles yet seen in a neurocyte. It is also said that the neurocytes are absent altogether during the incubation period, though the nervous system is already then infectious.

In dogs deceased from hydrophobia the Negri neurocytes are found in 98 to 99 per cent. Even in the few exceptional cases it is said that changes can always be detected in the plexiform and Gasserian ganglia, consisting of atrophy of the cells and invasion by the endothelium of the capsule. This is not specific to rabies, but is a valuable adjuvant to the Negri bodies as a means of diagnosis, and is always to be regarded as suspicious. These pathological discoveries have not yet been put to any practical application in the prevention and treatment of the disease, except in so far as they facilitate the diagnosis in the case of a suspected dog.

* Frosch: *Kolle und Wassermann's Handbuch der Micro-organismen*, 1907.

